# Stinging Trichomes in Apocynaceae and Their Evolution in Angiosperms

**DOI:** 10.3390/plants10112324

**Published:** 2021-10-28

**Authors:** Maria Camila Medina, Mariane S. Sousa-Baena, Natalie do Valle Capelli, Raquel Koch, Diego Demarco

**Affiliations:** Departamento de Botânica, Instituto de Biociências, Universidade de São Paulo, São Paulo 05508-090, SP, Brazil; camiliny@usp.br (M.C.M.); m.sousabaena@gmail.com (M.S.S.-B.); na.capelli@gmail.com (N.d.V.C.); raquelkoch@ib.usp.br (R.K.)

**Keywords:** glandular trichomes, plant defense, evolutionary convergence, anatomy, secretion, Apocynaceae

## Abstract

Stinging trichomes are rare in plants, occurring only in angiosperms, where they are reported for a few genera belonging to six families. Although there is no report of stinging trichomes in Apocynaceae, previous fieldwork collections of *Fischeria* and *Matelea* caused us a mild allergic reaction on the skin when we contacted the dense indumentum of the plants. This fact associated with the well-known presence of glandular trichomes with acute apex in both genera raised suspicions that stinging trichomes could be present in the family. Hence, this study aimed to investigate the likely occurrence of stinging trichomes in *Fischeria* and *Matelea*. We analyzed vegetative shoots and leaves of *Fischeria stellata* and *Matelea denticulata* through the usual procedures of light and scanning electron microscopy. We also performed several histochemical tests to investigate the chemical composition of trichome secretion. We detected that glandular trichomes occur throughout the surface of the leaf and stem. They are multicellular, uniseriate with an apical secretory cell, which has a dilated base and a needle-shaped apex. The secretion is compressed into the acuminate portion of the apical cell by a large vacuole, and crystals are deposited in the cell wall in a subapical position, providing a preferential site of rupture. The secretion, composed of amino acids and/or proteins, is released under mechanical action, causing skin irritation. Based on our detailed morphological and anatomical analyses, and in the functional aspects observed, we concluded that the glandular trichomes in *Fischeria* and *Matelea* can indeed be classified as stinging. Thus, Apocynaceae is the seventh family for which this type of trichome has been reported. We also compiled information on stinging trichomes in all families of angiosperms. Their phylogenetic distribution indicates that they have evolved at least 12 times during angiosperm evolution and may represent an evolutionary convergence of plant defense against herbivory.

## 1. Introduction

Flowering plants have several types of internal and external secretory structures for protection against herbivory. The first secretory structures to evolve were simple, consisting of single cells, e.g., idioblasts and laticifers. More complex internal structures, such as secretory ducts and cavities, appeared later in the evolutionary history of angiosperms. Apparently, the glandular trichomes evolved more recently. They have more complex secretory processes and dynamics of interaction with the environment as they are external structures [1,2]. Among glandular trichomes, the stinging ones stand out for their type of defense function against herbivory. These trichomes are rare, found in only a few angiosperm families, and their secretion is composed of a myriad of chemical substances [3].

Stinging trichomes are able to puncture the skin through their needle-shaped apical cells that have stiffened walls. When the tip of the trichome is broken, its contents are injected under the skin [4,5]. The secretion produces an allergic reaction in the skin (dermatitis), causing various symptoms from a mild irritation to death, depending on the plant species and contacting animal involved [1,5,6].

These trichomes have restricted occurrence, traditionally described as occurring in four families of eudicots: Euphorbiaceae, Hydrophyllaceae, Loasaceae and Urticaceae [5,6]. With the APG IV [7] update, which included changes in Boraginales, genera with stinging trichomes are now also placed in two additional families, i.e., Heliotropiaceae and Namaceae, resulting in six families possessing such a trait. In those families, they are usually comprised of an elongated secretory cell set on a multicellular pedestal. The secretory cell has a round basal portion and an acuminate apical portion that terminates with a needle-like tip [5]. However, two species of Apocynaceae from the Atlantic Rainforest are also called “nettle” by some local dwellers and caused skin irritation during fieldwork performed during our previous studies, thus indicating the possible existence of stinging trichomes in this family.

Only glandular trichomes have been described in Apocynaceae, where they are rare and reported for several genera of Asclepiadoideae: *Araujia*, *Cynanchum* (“*Sarcostemma*”), *Dischidia*; *Fischeria*, *Gongronema*, *Gonolobus*, *Marsdenia* and *Matelea* [8,9,10,11,12,13,14]. Particularly, *Fischeria* and *Matelea*, from the subtribe Gonolobinae, are the only genera in Apocynaceae that present a mixed indumentum consisting of short and long non-glandular trichomes and short glandular trichomes [9,11,12,13,14]. These glandular trichomes of *Fischeria* and *Matelea* have been described as containing an apical cell with an expanded base and a short apiculum, thus morphologically resembling stinging trichomes [11]. Recent research has shown that most genera of Gonolobinae (Asclepiadeae, Asclepiadoideae) have glandular trichomes, with a few exceptions [15,16,17]. However, none of them have been described as stinging trichomes. Hence, to elucidate the nature of these trichomes, we investigated the structure and distribution, as well as the composition of the secretion, of the glandular trichomes in *Fischeria stellata* and *Matelea denticulata*, discussing the results in terms of their possible function. We also review the occurrence and distribution of stinging trichomes in angiosperms.

## 2. Results

**Figure 1:**
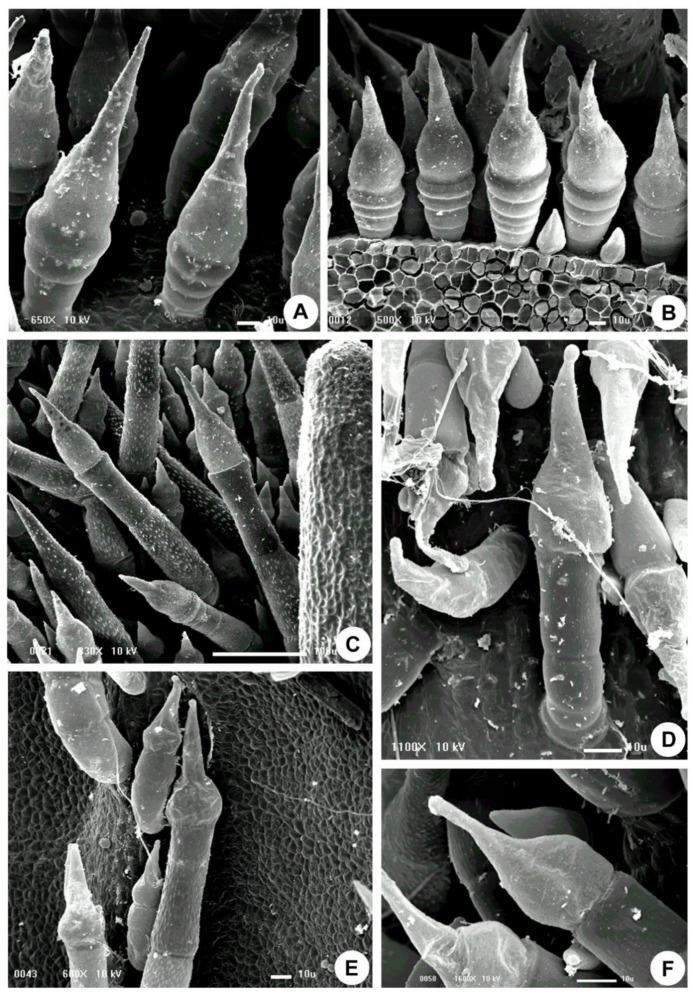
Scanning electron micrographs of the stinging trichomes of Apocynaceae. (**A**–**C**) *Fischeria stellata*. (**D**–**F**) *Matelea denticulata* (**A**,**D**) Stem. (**B**,**C**,**E**,**F**) Leaf.

**Figure 2:**
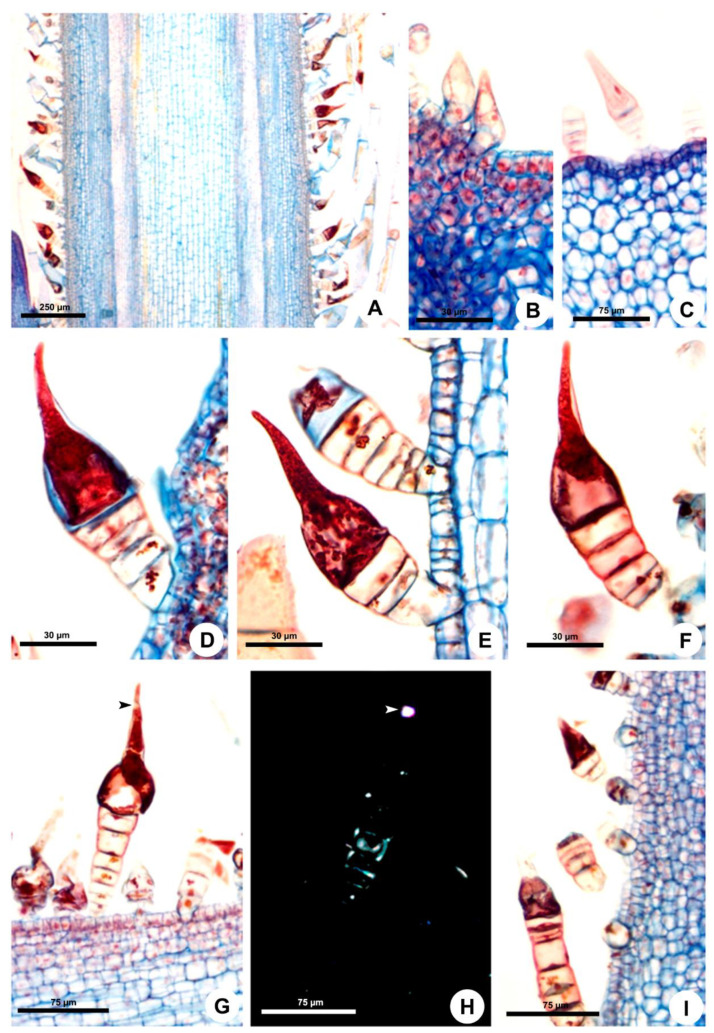
Ontogeny and structure of the stinging trichomes of *Fischeria stellata*. (**A**,**B**,**D**–**I**) Longitudinal sections. (**C**) Cross section. (**A**) Trichomes on the young stem. (**B**,**C**) Origin of the stinging trichomes on leaf primordia. (**B**) and primary stem (**C**). (**D**) Beginning of secretory activity. Note the dense aspect of the cytoplasm. (**E**) Secretory vesicles in the cytoplasm. (**F**) Mature trichome with vacuole in the basal region of the secretory cell and cytoplasmic contents in its acuminate region. (**G**,**H**) Mature stinging trichomes. Note the crystals (arrowhead) and the stalk with secondary walls evidenced by polarized light (**H**). (**I**) Stinging trichomes with the apex broken.

**Figure 3:**
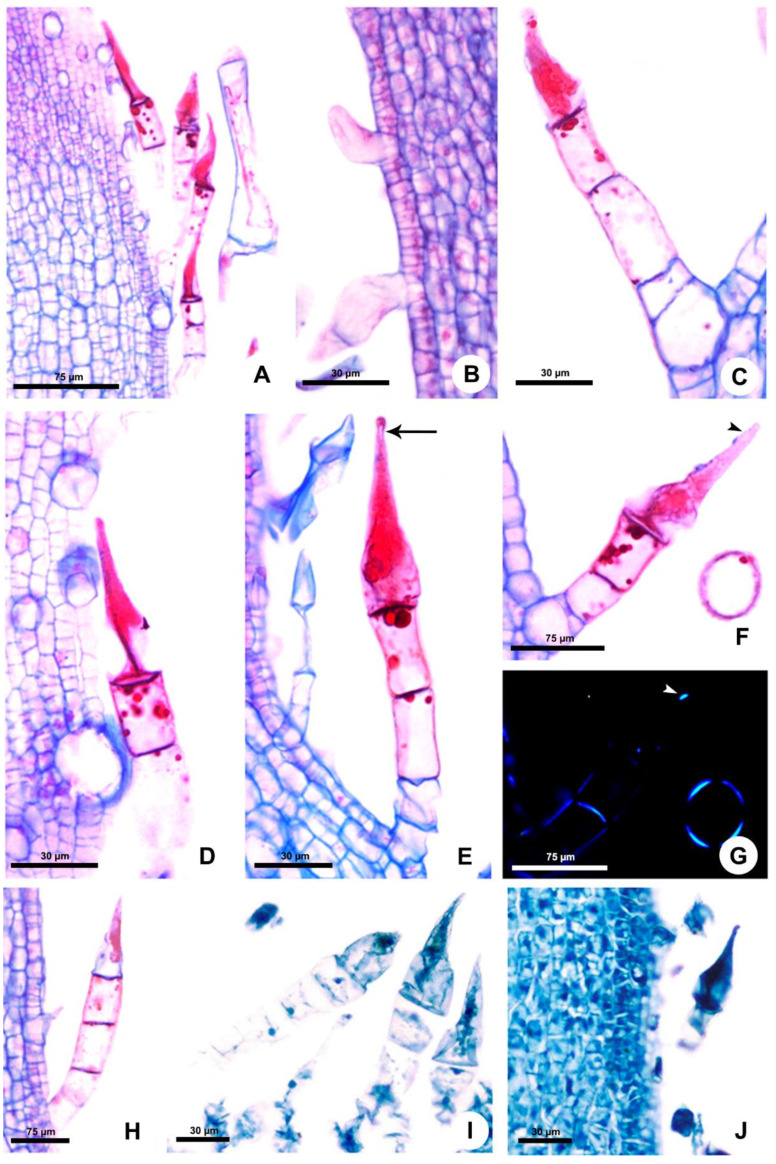
Origin, structure and histochemistry of the stinging trichomes of Apocynaceae. Longitudinal sections. (**A**–**H**,**J**) *Matelea denticulata.* (**I**) *Fischeria stellata*. (**A**) Trichomes on young leaf. (**B**) Origin of the stinging trichomes on leaf primordium. (**C**) General view of the stinging trichome. (**D**,**E**) Trichomes with the vacuole in the basal portion of the secretory cell and the cytoplasmic contents in the apical acuminate portion. Note the constriction (arrow) below the rounded apex (**E**). (**F**,**G**) Mature stinging trichome. Note the crystals (arrowhead) and the stalk with secondary walls evidenced by polarized light (**G**). (**H**) Stinging trichome with the tip broken, devoid of most part of its secretion. (**I**,**J**) Detection of proteins with aniline blue black.

In *Fischeria stellata* E.Fourn. and *Matelea denticulata* (Vahl) Fontella & E.A. Schwarz, the entire surface of the stem and leaves are covered by an indumentum composed of long, multicellular, and uniseriate non-glandular trichomes and short stinging trichomes. The stinging trichomes are multicellular, uniseriate with an apical secretory cell with an enlarged base and an acuminate upper portion (needle-shaped) (Figure 1, Figure 2 and Figure 3). They are brownish in fresh specimens and are easily distinguished from the others.

### Stinging Trichomes

They begin to develop from the protoderm of the second shoot node on the stem and leaf primordia (Figure 2A–C and Figure 3A,B). The trichomes continue to be produced throughout the development of these organs. The indumentum is dense in all developmental stages of stems and leaves (Figure 1B–D, Figure 2A and Figure 3A). The stinging trichomes can be recognized from the beginning of its formation since the secretory cell is the first to differentiate, becoming conical in the meristematic phase (Figure 2B,C and Figure 3B).

At maturity, in the third node of the shoot, the trichome stalk lignifies, providing a mechanical resistance to the gland (Figure 2G,H and Figure 3F,G). The trichome stalk is composed of three to eight cells in *Fischeria* (Figure 2D–I) and three cells in *Matelea* (Figure 3C–H). In *Fischeria*, the stalk generally exhibits a gradual increase in diameter towards the apex (Figure 2D–G). Conversely, in *Matelea*, the basal cell (or foot cell) is the wider one, followed by the stalk, composed of a narrow cell and two elongated cells containing intensely stained droplets (Figure 3C,E,F).

In the early secretory activity, the entire cytoplasm of the apical secretory cell is intensely stained (Figure 2D and Figure 3C). Subsequently, several vesicles are observed (Figure 2E), and a vacuole is formed at the cell base, compressing most of the cytoplasm contents into the conical upper portion (Figure 2F and Figure 3D,E). The large vacuole keeps the secretion under pressure in the needle-shaped apex, favoring the ejection of the secretion when the trichome tip is broken. Crystals are produced in the dilated cell base and transferred to a subapical position where they are deposited in the wall, making this region more fragile and prone to rupture by mechanical action (Figure 2G,H and Figure 3F,G). The trichomes of both species have a similar mechanism for releasing the secretion through apex rupture at the subapical crystal zone, with a large number of trichomes being found with the apical portion broken, devoid of most of their contents (Figure 2I and Figure 3H).

The trichomes of *Matelea* have a constriction below the rounded apex (Figure 1D and Figure 3E), whereas the trichomes of *Fischeria* have an acute apex, usually without constriction (Figure 1A,B and Figure 2D–G). The histochemical analysis showed that the trichome secretion is composed exclusively of amino acids and/or proteins in both genera (Table 1).

## 3. Discussion

Our study is the first to report the occurrence of stinging trichomes in Apocynaceae and in order Gentianales. In *Fischeria stellata* and *Matelea denticulata*, they cover the entire surface of the leaves and stems. The only studies focusing on structural aspects of glandular trichomes in Apocynaceae are from Solereder [8], who mentioned the trichomes of *Dischidia* as being unicellular and mucilaginous, and from Stevens [11], who described the glandular trichomes of *Matelea* as being smaller than the non-glandular ones, with a short stalk, an inflated middle portion, and a short apiculum.

### 3.1. Structure

The morphology of the stinging cell of the trichomes of *Fischeria* and *Matelea* resemble those described for other families [18,19]. This new stinging trichome is distinguished from the others by having a stalk since trichomes of the other families have an elongated stinging cell directly borne on the pedestal [5,6]. Besides Apocynaceae, these secretory structures are present only in Euphorbiaceae, Urticaceae, Hydrophyllaceae, Namaceae, Heliotropiaceae and Loasaceae [5,7] and likely evolved independently in these families. Nevertheless, trichome morphology and mechanism of secretion release is similar in all species. The only structural variation reported was observed in *Dalechampia* and *Tragia* (Euphorbiaceae), in which the stinging cell has a subprotodermal origin, unlike other species where its origin is protodermal. Additionally, in both genera, the trichomes have a crystal in the tip of the stinging cell that is forced out upon contact, puncturing the skin [5,19]. According to the literature, this structure has not been observed in other species, nor in the Apocynaceae examined in this study [5,6,19]. These seven families are restricted to the core eudicots and belong to orders from Rosids ((Malpighiales (Euphorbiaceae) and Rosales (Urticaceae)) and Asterids (Boraginales (Hydrophyllaceae, Namaceae and Heliotropiaceae), Cornales (Loasaceae), and Gentianales (Apocynaceae) [7]). In all these families, stinging trichomes are described as having a needle-shaped stinging cell with a constriction just below the apex and a bulbous cell base. This morphology is also observed in *Matelea*, which is similar to that observed in *Cnidoscolus*, *Loasa*, *Urera*, *Urtica* and *Wigandia* [5,18]. In Urticaceae, the apex breaks off upon contact, penetrating the skin and injecting its contents similar to a hypodermic needle [5,18]. This is one of the reasons why contact with stinging trichomes causes allergic reactions and the ability of puncture is apparently linked to the presence of silica in the trichome cell wall [20,21]. The presence of crystals deposited just below the apex is also important to create a potential rupture point in the cell wall, which favors the breakage of the apex, as observed in *Fischeria*, *Matelea* (this study) and other stinging trichomes [5]. Calcium phosphate and calcium carbonate were additional biominerals reported as present in the cell walls of stinging trichomes [21,22,23,24].

### 3.2. Function

Several studies of animal–plant interactions [25] have shown that leaf stinging trichomes of other species produce secretions that can cause reactions, such as the death of Lepidoptera larvae, itching in some mammals, as well as pain in humans, due to their defensive chemicals [3,26,27,28,29]. In some cases, the trichomes can puncture the body of insects, also acting also as a physical defense [30]. This is due to a diversity of toxic chemicals stored in the stinging trichomes. These components can range from a few to many depending on the species and may cause pain or irritation on the skin in humans [31,32]. Historically, Urticaceae have been the most studied family, with *Urtica dioica* being the species with the larger number of toxic chemicals described [5]. Common substances found in stinging trichomes of Urticaceae representatives are formic acid, acetylcholine, histamine, serotonin, alkaloids, acetic acid, among others [5,6]. In a study performed in *Laportea moroides*, acetylcholine, histamine and 5-HT (serotonin) were identified in stinging trichomes extract [4]. These components had already been observed in *Urtica dioica* [33]. In addition, 5-HT has also been identified in stinging trichomes of *Cnidoscolus texanus* from the family Euphorbiaceae [34].

In particular, stinging trichomes of species of Namaceae and Hydrophyllaceae have been investigated for the main presence of phenolic constituents. As a result, a complex mixture of methoxylated flavones and derivates of both farnesylhydroquinone and 3-farnesyl-p-hydroxybenzoic acid, which showed a strong dermatitis allergic effect, was identified in *Turriculia parryi* (Namaceae) [35]. In particular, a series of natural products called “phacelioids,” composed of geranylated or farnesylated 1,4-benzoquinones and hydroquinones, were identified in the stinging trichomes of *Phacelia* (Hydrophyllaceae) and *Wigandia* (Namaceae) as well as *Turriculia*. “Phacelioids” were shown to cause severe dermatitis upon contact with the plant [35,36,37]. Our tests for phenolic compounds were negative, revealing that the stinging substances of the trichomes of *Fischeria* and *Matelea* must be composed of other types of chemicals. Nevertheless, it is important to emphasize that some chemical components require highly sensitive analytical methods to be detected [31].

Among the histochemical tests we performed for *Fischeria* and *Matelea*, the only one with positive results was for protein/amino acids detection. However, in order to unravel whether the protein/amino acids histochemically detected are responsible for the trichome stinging properties it is necessary to perform analytical chemical studies. Interestingly, a recent study showed that *Dendrocnide excelsa* and *D. moroides*, from the family Urticaceae, produce toxic miniproteins that bear characteristics similar to some neurotoxins found in spiders and cone snail venoms [38].

In addition, it is not clear whether the toxins are produced exclusively in the stinging trichomes or if there exists some participation of the neighboring cells, with posterior transport to the stinging trichome, where they are finally stored [31]. We did not find any anatomical evidence in the species studied herein that suggest that non-glandular cells participate in the production of stinging substances. Such substances have biological value and can be used for medicinal purposes, thus being of economic interest [35].

### 3.3. Occurrence and Evolution of Stinging Trichomes

**Figure 4:**
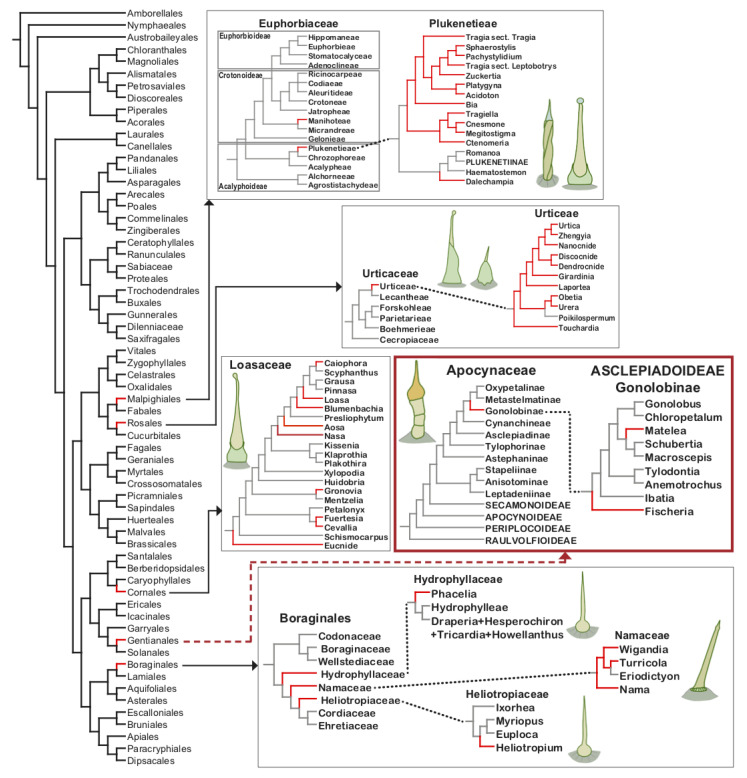
Simplified phylogenetic trees of the seven families that possess stinging trichomes, as well as of the more recently circumscribed order Boraginales showing the position of Hydrophyllaceae, Heliotropiaceae and Namaceae. Taxa where stinging trichomes occur are pointed out in red. Schematic drawings illustrate the trichome type found in the different families. Phylogenetic trees based on Huang et al. [39] and Wu et al. [40] for Urticaceae, Wurdack et al. [41] and Cardinal-McTeague and Gillespie [42] for Euphorbiaceae, Castillo et al. [43] and Hufford et al. [44] for Loasaceae, Mangelsdorff et al. [45] and Nazar et al. [46] for Apocynaceae, Hasenstab–Lehman [47] for Boraginales, Vasile et al. [48] for Hydrophyllaceae and Namaceae, and Hilger and Diane [49] for Heliotropiaceae.

The phylogenetic analysis of the position of families having stinging species (Figure 4), including the infrafamilial taxa (tribes; Table 2), suggests that the stinging trichomes have evolved independently at least 12 times during the evolution of eudicotyledons, being a good example of convergent evolution. Moreover, not all genera belonging to these seven families are stinging, with the characteristic being restricted to one or few tribes (Table 2). In Urticaceae, stinging trichomes evolved once in the tribe Urticeae. In Euphorbiaceae two evolutions occurred, in *Dalechampia* and in a large clade containing *Bia*, *Cnesmone* and *Tragia* among others (Figure 4). In Loasaceae it is possible that nine independent evolutions occurred, however the fact that closely-related stinging genera, i.e., *Aosa*, *Blumenbachia*, *Caiophora*, *Loasa*, and *Nasa*, are placed in the same monophyletic group (Figure 4) might suggest a single evolution of the character in the ancestor node from which *Nasa* and the remaining species diverged, resulting then in four independent evolution in the family. In the order Boraginales, a single evolution occurred in the families Hydrophyllaceae, Namaceae and Heliotropiaceae. In Apocynaceae two independent evolution occurred, in *Fischeria* and *Matelea*, species studied in this work. Hence, as the species containing stinging trichomes are phylogenetically closely related within tribes or subfamilies, future studies to investigate trichome development and evolution in such lineages may reveal if they carry phylogenetic and taxonomic value. It is also worthwhile to investigate other genera, close to *Fischeria* and *Matelea*, in Apocynaceae, as it is possible that stinging trichomes may be more widespread in the family than previously thought.

#### 3.3.1. Rosales

Stinging trichomes are structures typically associated with species of Urticaceae [6], which is the only family in the order Rosales with this type of trichome. More precisely, these trichomes occur only in Urticeae and may be a synapomorphy of the tribe, which has been hypothesized to promote species diversification [50]. Urticeae have 12 genera, of which 11 have stinging trichomes (Table 2). *Poikilospermum* is the only genus that does not have stinging trichomes, possibly due to secondary loss in the genus. The absence of stinging trichomes might be correlated with the habit of the genus, which is the only one composed of woody climbers in the tribe [34]. The systematic position of *Poikilospermum* has been considered quite controversial because its species have transitional features between Moraceae and Urticaceae [51]. Hence, it is possible that a reassessment of the phylogenetic relationships within the tribe reveals stinging trichomes are a synapomorphy of Urticeae [51]. The possibility is reinforced by the fact that the genus *Gyrotaenia* had been originally described as belonging to tribe Urticeae and having stinging trichomes [5]; however, Kim et al. [34] describe the genus as having no stinging trichomes. It has recently been proposed that *Gyrotaenia* is closer to tribe Elatostemateae than to Urticeae, considering the absence of stinging trichomes and the occurrence of female flowers with two tepals [40,50]. Both characters are important for the circumscription of Urticeae [50]. Our analysis indicates that the stinging trichomes evolved once in Urticaceae.

#### 3.3.2. Malpighiales

Stinging trichomes occur only in Euphorbiaceae. From the four subfamilies, only Acalyphoideae and Crotonoideae have representatives with stinging trichomes (Table 2). Most of them belong to the tribe Plukenetieae (Acalyphoideae). This tribe comprises three subtribes: (1) Tragiinae are the most genera-rich subtribe and have been characterized by the presence of abundant stinging trichomes [42]; (2) Dalechampiinae are a monogeneric subtribe. It is the most basal among the three subtribes and has been considered closely related to Tragiinae due to the presence of stinging trichomes [52]; (3) Plukenetiinae are the most apical tribe and do not have stinging trichomes (Figure 4). Thus, it seems that stinging trichomes evolved once in Plukenetieae (Acalyphoideae), being lost in Plukenetiinae. Only one genus of Crotonoideae has stinging species. This subfamily comprises 13 tribes, but only *Cnidoscolus* (tribe Manihoteae) has been described as having stinging trichomes (Table 2). Considering the current phylogenetic hypothesis of Euphorbiaceae (Figure 4), the stinging trichomes evolved independently twice within the family.

#### 3.3.3. Boraginales

Boraginales are the only order that has more than one family with stinging species. It is composed of 11 families [53], from which three families, i.e., Hydrophyllaceae, Heliotropiaceae and Namaceae, present species with stinging trichomes (Table 2).

**Table 2:** Distribution of stinging trichomes in angiosperms.

Family	Subfamily	Tribe	Genus	References
Apocynaceae	Asclepiadoideae	Asclepiadeae	*Fischeria*	this study
			*Matelea*	
Namaceae	-	-	*Nama*	[54,55]
			*Turriculia*	[35,36]
			*Wigandia*	[5,28,54,56]
Hydrophyllaceae	-	-	*Phacelia*	[22,35,54,57]
Heliotropiacae	-	-	*Heliotropium*	[58]
Euphorbiaceae	Acalyphoideae	Plukenetieae	*Acidoton*	[6,42]
			*Bia*	[42]
			*Cnesmone*	[5,6,33]
			*Ctenomeria*	[42]
			*Dalechampia*	[5,6,59]
			*Megistostigma*	[42]
			*Pachystylidium*	[5,31]
			*Platygyna*	[5,42]
			*Sphaerostylis*	[5,31,60]
			*Tragia*	[5,19,31,32,59,61]
			*Tragiella*	[5]
			*Zuckertia*	[42]
	Crotonoideae	Manihoteae	*Cnidoscolus*	[5,62,63,64,65]
Loasaceae	Gronovioideae	-	*Cevallia*	[66]
			*Fuertesia*	[67]
			*Gronovia*	[66]
	Mentzelioideae	-	*Eucnide*	[68]
	Loasoideae	Loaseae	*Aosa*	[21,23]
			*Blumenbachia*	[5,21,69]
			*Caiophora*	[21,22,69,70]
			*Loasa*	[21,22,69]
			*Nasa*	[21,23]
Urticaceae	-	Urticeae	*Dendrocnide*	[38,50]
			*Discocnide*	[50]
			*Girardinia*	[5,50]
			*Gyrotaenia*	[5]
			*Hesperocnide*	[5,50]
			*Laportea*	[5,50]
			*Nanocnide*	[5,50]
			*Obetia*	[5,50]
			*Urera*	[5,50]
			*Urtica*	[5,22,71]
			*Zhengyia*	[50]

Note. (-) Not applicable.

Hydrophyllaceae have 12 genera, of which only *Phacelia*, the largest and most diverse genus of the family (ca. 210 spp. out of 250) [7], is described as having stinging trichomes. The genus is monophyletic with many species having glandular trichomes [57] but not all have been described as possessing stinging trichomes [72,73].

Namaceae were segregated from Hydrophyllaceae and comprise four genera (*Eriodictyon*, *Nama*, *Turricula* and *Wigandia*), three of which have stinging trichomes (Table 2). The absence of stinging trichomes in *Eriodictyon* might be due to secondary loss as the genus is apical in the family phylogeny (Figure 4).

Heliotropiaceae, previously recognized as a subfamily of Boraginaceae, comprise four genera. The most species-rich is the paraphyletic *Heliotropium*, which has been described as having stinging trichomes [49]. More specifically, an anatomical analysis of *Heliotropium* showed that three (*H. digynum*, *H. strigosum* and *H. subulatum*) of the four species analyzed have stinging trichomes [58]. The other genera *Euploca*, *Ixorhea* and *Myriopus* were described as not having stinging trichomes [49,53]. However, it is possible to observe leaf trichomes similar to the stinging ones in a picture of *Myriopus* embedded in a study of foliar anatomy, although the authors have concluded that such trichomes were absent [74]. Thus, it is likely that a detailed analysis of the genus would reveal stinging trichomes in its representatives. Considering that Heliotropiaceae is a sister group of Hydrophyllaceae and Namaceae, it is possible that stinging trichomes evolved once in Boraginales, with reversals in Cordiaceae and Ehretiaceae.

#### 3.3.4. Cornales

The order Cornales consists of seven families, from which only Loasaceae have stinging trichomes. The family is divided in three subfamilies [75], in which this type of trichome occurs in all of them (Table 2). Loasoideae consist of three tribes: (1) Loaseae are the most apical tribe and have three genera with stinging trichomes (*Blumenbachia*, *Caiophora* and *Loasa*) (Figure 4); (2) Gronovioideae have four genera [76], with only one genus (*Petalonyx*) lacking stinging trichomes, and (3) Mentzelioideae have three genera, with only one (*Eucnide*) presenting stinging trichomes (Figure 4).

#### 3.3.5. Gentianales

Our results showed that *Fischeria* and *Matelea* have stinging trichomes. These genera are placed in the subfamily Asclepiadoideae, tribe Asclepiadeae and subtribe Gonolobinae (Table 2). Asclepiadoideae are the largest subfamily of Apocynaceae, with various tribes and subtribes. Notably, the subtribe Gonolobinae is the only group in which an annular corona occurs, which is a highly derived corona structure, in addition to glandular hairs in some representatives, a rare feature in the family [9,77,78,79]. Although *Fischeria* and *Matelea* are placed in the subtribe Gonolobinae [77] and share various morphological characteristics [9,12], a recent phylogenetic study shows these genera are not sister clades [45], indicating two independent evolutions of stinging trichomes in Apocynaceae.

## 4. Materials and Methods

*Fischeria stellata* (D. Demarco 58; D. Demarco 60) and *Matelea denticulata* (D. Demarco 37; D. Demarco 38) were collected in Ubatuba, São Paulo, Brazil. Vouchers of the individuals analyzed in this study were deposited in the UEC Herbarium (Universidade Estadual de Campinas).

For the anatomical study, vegetative branches of *Fischeria* and *Matelea* were fixed in FAA (formalin, acetic acid, alcohol) for 24 h [80], BNF (buffered neutral formalin) in sodium phosphate buffer 0.1 M pH 7.0) [81] and FSF (ferrous sulfate in formalin) [80] for 48 h and stored in ethyl alcohol 70%. Apical shoots were isolated, dehydrated in a tertiary butyl alcohol series [80], embedded in Paraplast (Leica Microsystems, Heidelberg, Germany), and transversely and longitudinally sectioned in a Microm HM340E rotary microtome (Microm, Walldorf, Germany). The sections were stained with astra blue and safranin (color index (C.I.) 50240) [82], and the slides were mounted in synthetic resin. Photomicrographs were taken using a Leica DMLB light microscope (Leica Microsystems, Wetzlar, Germany).

For micromorphological analysis, shoots and leaves fixed in FAA were isolated, dehydrated in an ethanol series, critical-point dried, mounted on stubs and coated with gold. The observations and recording of images were performed using a Jeol JSM 5800 LV 10 kV scanning electron microscope (Jeol, Tokyo, Japan) with a digital camera attached.

For histochemical analysis, different treatments were performed to highlight the major chemical classes of the constituents of the trichome secretion: ruthenium red for acidic mucilage [83], tannic acid and ferric chloride for mucilage [84], PAS reaction (periodic-acid-Schiff; pararosaniline C.I. 42500) for carbohydrates [85], aniline blue black (C.I. 20470) for proteins [86], Sudan black B (C.I. 26150) and Sudan IV (C.I. 26105) for lipids [87], Nile blue (C.I. 51180) for acidic and neutral lipids [88], copper acetate and rubeanic acid for fatty acids [89,90], and ferric chloride for phenolic compounds [80]. The slides were mounted in a glycerin–gelatin medium. The controls were performed according to Demarco [91].

## 5. Conclusions

This is the first report of stinging trichomes for Apocynaceae and for Gentianales as a whole. Hence, stinging trichomes are currently described in members of seven distantly-related angiosperm families, indicating such a secretory structure evolved multiple times during the evolution of plants. We classified trichomes of *Fischeria stellata* and *Matelea denticulata* as stinging due to their morphology, mechanism of secretion release, and composition of the secretion that causes contact dermatitis. Interpreting the occurrence of stinging trichomes in the diverse families indicates that they evolved at least 12 times during angiosperm evolution and may represent an evolutionary convergence of plant defenses against herbivory. The presence of stinging trichomes is likely a synapomorphy of the tribe Urticeae from Urticaceae, likely evolving in the tribe ancestor with a reversal of the character in *Poikilospermum*. In the other families with stinging trichomes (Apocynaceae, Euphorbiaceae, Hydrophyllaceae, Namaceae, Heliotropiaceae and Loasaceae), these structures apparently evolved independently in several lineages. The unique mechanism of secretion injection within the skin together with the complex combination of substances composing the secretion are likely responsible for the stinging properties of these trichomes. Such studies of the subject are scarce and might shed light on the evolution of stinging trichomes.

## Figures and Tables

**Table 1:** Histochemical tests applied to identify the major classes of metabolites of the stinging trichome secretion in *Fischeria stellata* (Fs) and *Matelea denticulata* (Md).

Histochemical Treatment	Detected Substance	Secretion	
		Fs	Md
Ruthenium red	acidic mucilage	−	−
Tannic acid and ferric chloride	mucilage	−	−
PAS reaction	carbohydrates	−	−
Ferric chloride	phenolic compounds	−	−
Formalin-ferrous sulphate	phenolic compounds	−	−
Aniline blue black	proteins	+ (Figure 3I)	+ (Figure 3J)
Sudan black B	lipids	−	−
Sudan IV	lipids	−	−
Nile blue	neutral and acidic lipids	−	−
Copper acetate and rubeanic acid	fatty acids	−	−

Note. + present; − absent.
